# Climate change: a priority agenda for health services

**DOI:** 10.1590/S2237-96222025v34e20252001.en

**Published:** 2025-05-26

**Authors:** Everton Nunes da Silva, Jorge Otávio Maia Barreto, Maria Auxiliadora Parreiras Martins, Wildo Navegantes de Araújo, Taís Freire Galvão

**Affiliations:** ¹ Universidade de Brasília, Faculdade de Ciências e Tecnologias em Saúde, Brasília, DF, Brazil; ² Fundação Oswaldo Cruz, Gerência Regional de Brasília, Brasília, DF, Brazil; ³ Universidade Federal de Minas Gerais, Faculdade de Farmácia, Belo Horizonte, MG, Brazil; ⁴ Universidade Estadual de Campinas, Faculdade de Ciências Farmacêuticas, Campinas, SP, Brazil

Extreme weather events have become increasingly frequent in different parts of the world, reaching magnitudes capable of causing significant harm to society - especially among those individuals in more vulnerable socioeconomic conditions _^^1^).^_ In this context, Brazil represents an emblematic case. In 2024, the state of Rio Grande do Sul was severely impacted by flooding, while a historic drought occurred in the Amazon region. In the same year, wildfires affected various biomes and both rural and urban populations across the country. Among the negative impacts of climate change, the effects on human health require greater understanding - both at the individual level, in terms of illness or reduced well-being, and at the collective health level, where climate change influences the response capacity of health systems, exacerbates inequities, and deepens failures in multisectoral coordination in formulating and implementing mitigation and adaptation measures, particularly to address extreme weather events.

Climate change has led to increases in respiratory diseases, mental health problems, malnutrition, food insecurity, infectious diseases - including arboviruses and other zoonoses - and other pressing public health problems ^2^. However, the currently available evidence has largely been generated in high-income settings ^3^, which may limit understanding of the health effects of climate events in contexts marked by poverty and other socioeconomic determinants. Additionally, certain population groups will face broader and more profound impacts during extreme weather events, such as individuals with mobility limitations, disabilities, older adults, pregnant women, and children ^4^. These groups remain under evaluated in studies examining the health effects of climate-related issues.

The emergence and spread of antimicrobial resistance (AMR) have been associated with climate change in studies conducted in health services in Europe ^5^ and the United States ^6^. AMR occurs when bacteria, viruses, fungi, and parasites no longer respond to treatments, leading to worse clinical outcomes and increasing the spread of hard-to-treat infections. The World Health Organization (WHO) considers AMR a public health priority due to its high morbidity and mortality burden. This issue was discussed at the 2024 United Nations General Assembly, where it was highlighted that AMR will cause even greater global suffering - particularly in low- and middle-income countries. In these countries, the consequences are especially concerning due to their limited capacity to respond to health crises ^7^. Although research on the relationship between climate change and AMR is still in its early stages, the findings are consistent, and further studies are needed to fill knowledge gaps, given the potential human, environmental, and economic impacts ^8^.

Health systems are also impacted by global warming. The most direct example is when facilities are damaged or destroyed by environmental disasters such as floods, landslides, or wildfires ^9^. Other issues affecting health service operations and linked to extreme climate events include staff absenteeism and presenteeism, loss of supplies, and interruptions in power, drinking water, and sewage systems ^10^. Additionally, climate events lead to increased demand for health care due to changing health needs and migration ^11^, placing greater pressure on local and national systems.

In this context, studies on the resilience of health systems are essential. For example, research should aim to identify and prioritize strategies to anticipate climate events and to enhance institutional and social capacities for rapid response, thereby ensuring the continued delivery of timely and quality health services ^12^. Furthermore, the health sector accounts for approximately 5% of global greenhouse gas emissions, making it necessary to implement mitigation plans within the sector ^13^.

Health inequities are exacerbated by extreme climate events. Populations living in vulnerable areas - often underserved by health services - tend to face even more restricted access, including to ongoing mitigation and adaptation actions ^14^. These populations are also more likely to incur catastrophic expenditures, which may further impoverish them, whether due to healthcare costs or other expenses such as repairing or rebuilding homes and livelihoods ^15^.

Considering this scenario, addressing the causes and consequences of climate change requires coordinated multisectoral efforts at global, national, and local levels. Internationally, a major opportunity arises with the 30th United Nations Climate Change Conference (COP30), to be held in Belém, Brazil, in November 2025. Beyond country-level commitments to develop and implement mitigation and adaptation strategies for global warming, increased financial investment in this area is anticipated. Multilateral funding sources for climate change remain scarce, particularly in Latin America ^14^.

At national and local levels, greater constructive interaction and coordination are expected within the country’s evidence ecosystem, bringing together producers and users of scientific knowledge, decision-makers, and organized civil society. This would help ensure that several types of evidence become accessible, valued, and effectively incorporated in a timely manner to address challenges posed by climate change. Key-stakeholders in this ecosystem include researchers and decision-makers from sectors such as health, environment, science and technology, civil protection, social assistance, and the productive sector. To foster effective linkages between knowledge and decision-making, it is necessary to rely on trustworthy, transparently and systematically generated information, supported by comprehensive, consistent, real-time information systems. Reliable data are a key element in measuring the effects of climate change and monitoring and evaluating the results of ongoing mitigation and adaptation policies.

*Epidemiologia e Serviços de Saúde: revista do SUS* (RESS), committed to the topic, highlights some articles published at RESS that enrich the debate, addressing topics such as the estimation of human and material damage due to disasters ^16^ - published in current volume 34 -, effects of climate change on the incidence of dengue ^17^, leptospirosis ^18^, diseases subject to compulsory notification ^19^, and health effects of dam disasters ^20^.

RESS encourages the academic and health service community to submit studies that analyze the effects of climate change on health, particularly research with implications for the Brazilian Unified Health System.
