# Supplementary material for: Climate change: a priority agenda for health services
Source: Epidemiol Serv Saude. 2025 May 26;34:e20252001. doi: 10.1590/S2237-96222025v34e20252001.en (PMC12105842; doi:10.1590/S2237-96222025v34e20252001.en)
Supplement: Translation

# Translation: Mudanças climáticas: uma agenda prioritária aos serviços de saúde

**DOI:** 10.1590/S2237-96222025v34e20252001.pt

**Authors:** Everton Nunes da Silva, Jorge Otávio Maia Barreto, Maria Auxiliadora Parreiras Martins, Wildo Navegantes de Araújo, Taís Freire Galvão

**Affiliations:** 1 Universidade de Brasília, Faculdade de Ciências e Tecnologias em Saúde, Brasília, DF, Brasil; 2 Fundação Oswaldo Cruz, Gerência Regional de Brasília, Brasília, DF, Brasil; 3 Universidade Federal de Minas Gerais, Faculdade de Farmácia, Belo Horizonte, MG, Brasil; 4 Universidade Estadual de Campinas, Faculdade de Ciências Farmacêuticas, Campinas, SP, Brasil

Os eventos climáticos extremos têm se tornado mais frequentes em diferentes partes do mundo e alcançado magnitude capaz de gerar danos importantes à sociedade, em especial àqueles indivíduos que vivem em condições socioeconômicas mais vulneráveis ^1^. Nesse cenário, o caso brasileiro é emblemático. Em 2024, o Rio Grande do Sul foi fortemente atingido pelas inundações, ao mesmo tempo que uma seca histórica ocorria na região amazônica. Nesse mesmo ano, as queimadas afetaram diferentes biomas e populações, rurais e urbanas, em quase todo o território nacional. Entre os impactos negativos das mudanças climáticas, os efeitos sobre a saúde das pessoas precisam ser mais bem compreendidos, tanto na dimensão individual do adoecimento, ou menor bem-estar social, como na dimensão da saúde coletiva, na qual as mudanças climáticas influenciam a capacidade de resposta dos sistemas de saúde, agravam iniquidades, e aprofundam falhas na coordenação multissetorial atinentes à formulação e implementação de medidas de mitigação e de adaptação, em especial para fazer frente aos eventos climáticos extremos.

As mudanças climáticas têm aumentado a incidência de doenças respiratórias, problemas de saúde mental, desnutrição, insegurança alimentar, doenças infeciosas - incluindo as arbovirose e outras zoonoses -, entre outros problemas prioritários de saúde coletiva ^2^. No entanto, as evidências hoje disponíveis foram obtidas em cenários de renda alta ^3^, o que pode limitar a compreensão sobre os efeitos dos eventos climáticos em doenças e condições de saúde relacionadas à pobreza e a outros determinantes socioeconômicos. Ademais, há grupos populacionais que sofrem consequências mais amplas e profundas nos eventos climáticos extremos, como aqueles indivíduos com limitações de mobilidade, incapacidades de diferentes naturezas, idosos, gestantes e crianças ^4^. Esses grupos têm sido pouco abordados em estudos concernentes aos efeitos das questões climáticas sobre a saúde.

O aparecimento e a disseminação de resistência microbiana foram associados a mudanças climáticas em estudos realizados em serviços de saúde na Europa ^5^ e nos Estados Unidos ^6^. A resistência microbiana ocorre quando bactérias, vírus, fungos e parasitas não respondem mais ao efeito dos medicamentos, levando à piora clínica e aumentando a disseminação de infecções difíceis de tratar. A Organização Mundial da Saúde (OMS) considera esse tema prioritário para a saúde pública, devido à sua elevada morbimortalidade. Essa pauta foi discutida na Assembleia Geral da Organização das Nações Unidas, realizada em 2024, ressaltando-se que a resistência microbiana causará ainda mais sofrimento global, particularmente em países de baixa e média renda. Nesses países, as consequências podem ser mais preocupantes, em função da baixa capacidade de resposta a situações de crise e agravos à saúde ^7^. Embora as pesquisas acerca da influência das mudanças climáticas sobre a resistência microbiana ainda sejam incipientes, os achados têm se mostrado convergentes, e novos estudos precisam ser realizados para investigar as lacunas do conhecimento, tendo em vista seu potencial impacto humano, ambiental e econômico ^8^.

Os sistemas de saúde também são afetados pelo aquecimento global. É o que se verifica quando os estabelecimentos são atingidos por desastres ambientais, que lhes causam danos ou destruição ^9^, tais como inundações, deslizamentos de terra ou queimadas. Outras questões que afetam o funcionamento dos serviços de saúde e estão relacionadas com eventos climáticos extremos incluem o absenteísmo e o presenteísmo de recursos humanos, a perda de insumos, interrupções no abastecimento de energia, água potável e sistemas de esgoto ^10^. Adicionalmente, os eventos climáticos provocam maior pressão na demanda por cuidados em saúde, devido a mudanças nas necessidades de saúde e a migrações ^11^, sobrecarregando os sistemas locais e nacionais com demandas emergentes.

Nesse contexto, os estudos sobre resiliência dos sistemas de saúde são essenciais. Por exemplo, estudos que busquem descobrir e priorizar estratégias de antecipação aos eventos climáticos e aumentar as capacidades institucionais e sociais para pronta resposta, a fim de se garantir o funcionamento das ações e serviços de saúde, com qualidade e em tempo oportuno ^12^. Ademais, o setor de saúde é responsável por cerca de 5% das emissões de gases do efeito estufa, sendo necessária também a adoção de planos de mitigação nessa área ^13^.

As iniquidades em saúde são agravadas pelos eventos climáticos extremos. Populações vivendo em áreas vulnerabilizadas, que muitas vezes são descobertas por serviços de saúde, tendem a ter seu acesso ainda mais limitado, inclusive às ações de mitigação e adaptação em curso ^14^. Estas populações também são mais propensas a incorrer em despesas catastróficas, que podem agravar seu empobrecimento, seja por gastos em saúde, seja em decorrência de gastos de outra natureza, como aqueles destinados à reparação ou reconstrução de suas moradias e retomada de suas vidas ^15^.

Diante desse cenário, o enfrentamento das causas e consequências das mudanças climáticas requer um esforço de concertação multissetorial, abrangendo os níveis global, nacional e local. Em termos internacionais, abre-se uma grande oportunidade com a 30ª Conferência das Nações Unidas sobre Mudanças Climáticas (COP30), a ser realizada em Belém, Brasil, em novembro de 2025. Além do comprometimento dos países em elaborar e implementar estratégias de mitigação e adaptação ao aquecimento global, espera-se maior aporte de recursos para essa área. Ainda são escassas as fontes de financiamento multilaterais voltadas para as mudanças climáticas, especialmente na América Latina ^14^.

Nos âmbitos nacional e local, são essenciais maior sinergia e melhor coordenação do ecossistema de evidências nacional, articulando-se produtores e usuários do conhecimento científico, tomadores de decisão e membros da sociedade civil organizada, para que as evidências de diferentes tipos possam se tornar acessíveis, valorizadas e úteis, e incorporadas oportunamente, diante dos graves problemas decorrentes da mudança climática. Os atores e artífices deste ecossistema incluem pesquisadores e tomadores de decisão de diferentes setores, como saúde, meio ambiente, ciência e tecnologia, defesa civil, assistência social e setor produtivo. Para que a articulação entre conhecimento e decisão seja virtuosa, é necessário contar com informação confiável, gerada de modo transparente e sistemático, com o suporte de sistemas de informação abrangentes, consistentes e em tempo real. Informação confiável é uma peça-chave para a mensuração dos efeitos das mudanças climáticas, bem como para a monitoração e avaliação dos resultados das políticas de mitigação e adaptação em andamento.

*A Epidemiologia e Serviços de Saúde: revista do SUS* (RESS), compromissada com a temática, destaca alguns artigos publicados na RESS que enriquecem o debate, versando sobre a estimativa de danos humanos e materiais devidos a desastres ^16^ - publicado no corrente volume 34 -, os efeitos das mudanças climáticas na incidência de dengue ^17^, leptospirose ^18^, doenças de notificação compulsória ^19^, e efeitos na saúde de desastres com barragens ^20^.

A RESS incentiva as comunidades acadêmica e de serviços de saúde a submeterem estudos que analisem os efeitos das mudanças climáticas na saúde, principalmente com investigações de implicações para o Sistema Único de Saúde.
